# Stakeholder perspectives around post-TB wellbeing and care in Kenya and Malawi

**DOI:** 10.1371/journal.pgph.0000510

**Published:** 2022-09-07

**Authors:** Sarah Karanja, Tumaini Malenga, Jessie Mphande, Stephen Bertel Squire, Jeremiah Chakaya Muhwa, Ewan M. Tomeny, Laura Rosu, Stephen Mulupi, Tom Wingfield, Eliya Zulu, Jamilah Meghji

**Affiliations:** 1 African Institute for Development Policy (AFIDEP), Lilongwe, Malawi; 2 Kenya Medical Research Institute, Centre for Public Health Research (KEMRI-CPHR), Nairobi, Kenya; 3 Tropical and Infectious Diseases Unit, Liverpool University Hospital NHS Foundation Trust, Liverpool, United Kingdom; 4 Faculty of Clinical Sciences & International Public Health, Liverpool School of Tropical Medicine, Liverpool, United Kingdom; 5 Department of Clinical Studies, Liverpool School of Tropical Medicine, Liverpool, United Kingdom; 6 Department of Medicine, Therapeutics, Dermatology and Psychiatry, Kenyatta University, Nairobi, Kenya; 7 Social medicine, infectious diseases and migration research group and WHO Collaborating Centre on Tuberculosis and Social Medicine, Department of Global Health Sciences, Karolinksa Institute, Solna, Sweden; 8 Department of Respiratory Medicine, Cambridge University Hospitals NHS Foundation Trust, Cambridge, United Kingdom; University of California San Francisco, UNITED STATES

## Abstract

**Background:**

There is growing awareness of the burden of post-TB morbidity, and its impact on the lives and livelihoods of TB affected households. However little work has been done to determine how post-TB care might be delivered in a feasible and sustainable way, within existing National TB Programmes (NTPs) and health systems, in low-resource, high TB-burden settings. In this programme of stakeholder engagement around post-TB care, we identified actors with influence and interest in TB care in Kenya and Malawi, including TB-survivors, healthcare providers, policy-makers, researchers and funders, and explored their perspectives on post-TB morbidity and care.

**Methods:**

Stakeholder mapping was completed to identify actors with interest and influence in TB care services in each country, informed by the study team’s local, regional and international networks. Key international TB organisations were included to provide a global perspective. In person or online one-to-one interviews were completed with purposively selected stakeholders. Snowballing was used to expand the network. Data were recorded, transcribed and translated, and a coding frame was derived. Data were coded using NVivo 12 software and were analysed using thematic content analysis. Online workshops were held with stakeholders from Kenya and Malawi to explore areas of uncertainty and validate findings.

**Results:**

The importance of holistic care for TB patients, which addresses both TB comorbidities and sequelae, was widely recognised by stakeholders. Key challenges to implementation include uncertainty around the burden of post-TB morbidity, leadership of post-TB services, funding constraints, staff and equipment limitations, and the need for improved integration between national TB and non-communicable disease (NCD) programmes for care provision and oversight. There is a need for local data on the burden and distribution of morbidity, evidence-informed clinical guidelines, and pilot data on models of care. Opportunities to learn from existing HIV-NCD services were emphasised.

**Discussion:**

This work addresses important questions about the practical implementation of post-TB services in two African countries, exploring if, how, where, and for whom these services should be provided, according to a broad range of stakeholders. We have identified strong interest in the provision of holistic care for TB patients in Kenya and Malawi, and key evidence gaps which must be addressed to inform decision making by policy makers, TB programmes, and funders around investment in post-TB services. There is a need for pilot studies of models of integrated TB care, and for cross-learning between countries and from HIV-NCD services.

## Background

### Burden of post-TB morbidity

The global burden of TB disease remains unacceptably high with an estimated 9.9-million incident cases of TB disease in 2020 [1]. However, the treatment success rate for those receiving first-line regimens was 86% in 2019 [1], and there are an estimated 155-million TB-survivors alive today [2].

It is increasingly clear many of these TB-survivors experience long-term morbidity even after treatment completion, including post-TB lung disease (PTLD), with abnormal spirometry and structural lung pathology seen in over a third of those successfully treated for pulmonary tuberculosis (PTB) [3–6], socioeconomic morbidity with difficulty recovering income and employment [7], and long-term psychological morbidity related to stigma and social isolation [8]. TB-survivors have mortality rates which are over three-times higher than TB naïve adults, even after successful treatment completion, with cardiovascular disease identified as a common cause of death [9, 10]. Individuals who have had a first episode of TB disease are at increased risk of recurrent TB disease, compared to the TB naïve population, due to relapse and reinfection [11, 12]. Modelling data suggest that the disability-adjusted life years (DALYs) incurred due to TB sequalae may match or even exceed those incurred during TB disease itself [13, 14].

### Existing context of care

In response to the growing evidence for a high burden of morbidity and mortality amongst TB-survivors, there have been calls from TB-affected groups, healthcare providers, and researchers for the development of clinical guidelines and programmatic standards for post-TB patient care [15–18]. However, there remain many barriers to implementation.

Firstly, the existing paradigm of TB care remains focused on improving diagnosis, treatment and survival during TB disease itself. National and international TB guidelines and targets do not include the long-term wellbeing of TB survivors, and post-TB morbidity is not routinely recorded and not prioritised within treatment programmes or clinical trials [15]. Secondly, post-TB morbidity includes multiple dimensions such as physical, psychological, social and economic wellbeing [8]. The relationships between these parameters are unclear, and there is a lack of robust evidence for interventions which improve patient outcomes across these parameters. Existing guidelines for post-TB care are mostly clinical, and are largely rooted in expert opinion only [18]. Thirdly, even as evidence for the types of support required by TB-survivors improves, there is a lack of pilot studies or implementation based work describing how these services might be delivered in a feasible and sustainable way, within existing National TB Programmes (NTPs) and health systems. This may be particularly challenging in low-resource, high TB-burden settings where resources are stretched, and it remains unclear how, when, and to whom post-TB services might be provided.

### Aims and objectives

In response to this lack of implementation data, we completed an 18-month programme of stakeholder engagement around post-TB care in Kenya and Malawi. The aim of this work was to inform the development of strategies for post-TB care within the region. The objectives were to identify and connect stakeholders in TB service delivery in Kenya and Malawi, to raise awareness of post-TB wellbeing amongst these stakeholders, to understand the existing context of care, and to explore beliefs and perspectives around post-TB morbidity and care. Findings of this stakeholder engagement work are presented here.

## Methods

This study was run as a partnership between The Liverpool School of Tropical Medicine (LSTM) and The African Institute for Development Policy (AFIDEP), in collaboration with the National Tuberculosis, Leprosy and Lung Disease Programme (NTLP) in Kenya, and the National Tuberculosis Control Programme (NTP) in Malawi (Feb 2020 to July 2021).

### Stakeholder mapping

An initial process of stakeholder mapping was completed at the start of the programme, to identify individuals with interest and influence in post-TB wellbeing in Kenya and Malawi. This was informed by the local, regional and international networks of the study team from LSTM and AFIDEP. In-country stakeholders included policy makers, parliamentarians, funders, researchers, health care workers, TB advocates and TB patients and survivors. Key international TB organisations were included to provide a global perspective. A snowballing approach was then used to identify further stakeholders, over the course of the study. The number of interviews completed was determined by the number of key stakeholders identified, and we did not specify a requirement for data saturation.

### Development of data collection tools

Three data collection tools were used to guide semi-structured interviews with different stakeholder groups (Appendix A in S1 File: TB survivors and patient advocates; Local & regional stakeholders; International stakeholders). These tools explored existing practices and perceived need for post-TB care, key evidence gaps, perceived barriers to implementation, and potential structure, content, and leadership of post-TB services. These topics were identified *a priori* by the study team as relevant to the post-TB agenda, based on their own experience and published literature, with iterative review over the course of data collection. Post-TB sequelae were broadly conceptualised as the multifactorial challenges faced by TB survivors, including physical, psychological, social and economic morbidity, and risk of recurrent TB disease.

### Stakeholder interviews

Key stakeholders identified in the mapping exercise were invited to participate in individual interviews. Interviews were conducted on a one-to-one basis, through phone calls, online or in-person meetings, in keeping with national COVID-19 guidelines and were conducted by researchers from AFIDEP (SK, TM) with previous experience of qualitative research and community engagement. Interviews were completed between October 2020 and March 2021. Participants were provided with a paper or electronic information leaflet about the study, and informed consent was taken either verbally with recording, or in writing. Interviews were conducted in English, Swahili or Chichewa at participant preference, and were audio-recorded and transcribed verbatim.

### Data coding and analysis

Interview transcripts were read by study authors (SK, TM, JMp, JMe). A coding framework was derived (Appendix B in S1 File), and the data were coded using Nvivo 12 software (SK, TM). Data were analysed using thematic content analysis. Coded data were reviewed and discussed regularly in order to identify key concepts, emerging themes, and determine data findings. Notes kept by team members, and records of team meetings were used to inform this analysis‥ Findings were discussed with the broader team at regular intervals to clarify these themes and findings, identify areas of uncertainty, and to inform ongoing stakeholder engagements.

### Data validation and exploration

Once key themes had been identified, virtual workshops were held in Kenya and Malawi, with a broad range of stakeholders invited, whether interviewed or not. International stakeholders were invited to attend the Kenyan workshop. Workshops were video recorded with verbal consent from all participants, and included break-out sessions to explore key themes emerging from interview data (Appendix B in S1 File). The recordings were shared with all participants with their consent. Workshops were not transcribed, but notes were taken by the study team during each session, and used to inform and advance our understanding of the data generated from individual interviews.

### Ethical approval

Formal ethics applications were submitted in Kenya, Malawi and to LSTM. Ethical approval was obtained from LSTM (20–064) for work in both countries and KEMRI for work in Kenya (KEMRI/RES/7/3/1). Ethical approval was waived by the National Health Sciences Research Committee in Malawi as the study focused on service design and development.

For confidentiality reasons, the term ‘Ministry of Health’ or MoH is used in this manuscript to describe quotes obtained from NTP or NCD Programme members from Kenya and Malawi.

## Results

In this section we describe the stakeholders who participated in this study, and data collected on the existing context and perceived need for post-TB care. We then describe key themes which emerged on the structure and delivery of post-TB services, and potential barriers to implementation.

### Stakeholder engagements

Forty-seven key multisectoral stakeholders were identified through stakeholder mapping, with 38 interviews completed with TB-survivors, healthcare workers, funders, policy-makers and researchers from Kenya, Malawi and relevant international organisations (Table 1). Nine of the stakeholders we invited to participate in the study did not respond to our invitation. The majority of patient advocates interviewed were themselves TB-survivors.

**Table 1:** Stakeholder interviews.

Stakeholder group	Malawi (n = 12)	Kenya (n = 20)	International (n = 6)
National TB Programme	2	3	
National NCD Programme	1	1	
Other government ministries	0	3	
Healthcare provider	4	6	
TB-survivor	1	1	
TB patient advocacy group	2	4	
In-country researcher	1	0	
In-country non-governmental organisation (NGO)	1	2	
Multi-lateral Organisation (Funding)	0	0	2*
Multi-lateral Organisation (TB policy)	0	0	4^†^

*The Global Fund, U.S. Agency for International Development (USAID)

^†^The International Union against Tuberculosis and Lung Disease, Stop TB Partnership, World Health Organisation Kenya, World Health Organisation Malawi

A total of 77 stakeholders were invited to the workshops: 34/43 invited stakeholders attended the Malawi workshop and 25/34 invited stakeholders attended the Kenya workshop, giving a total of 59 participants. Workshops lasted three hours long for each country.

### Reported context of care

Neither Kenya nor Malawi have active programmes for post-TB care. However, a technical working group for PTLD was established within the Kenyan NTLP in 2019, to “coordinate, operationalize, and entrench” PTLD activities. The next version of the integrated NTLP TB treatment guidelines is expected to include a chapter focused on post-TB lung health. Potential reasons given for the focus on this agenda include that two Kenyan NTLP delegates attended an international post-TB symposium in South Africa in 2019 [8], and that a recent review of the chest X-rays captured in the Kenyan TB Prevalence survey in 2015–16 demonstrated a high prevalence of post-TB lung pathology [19]. However, it is not clear whether these efforts were the cause of, or a response to, the growing interest in this field. Prior to this study, there was no clear working group for post-TB health within either the national TB or NCD programmes in Malawi, and no mention of post-TB care within national guidelines. However, interest in developing a comparable Malawian post-TB working group was expressed in the Malawian workshop held at the end of this programme of work.

### Perceived need for post-TB services (Table 2)

**Table 2:** Direct participant quotes: Perceived need for post-TB services.

Theme	Sub-theme	Quotes
Not a priority at present	Need to focus on public health	“There are more pressing issues. It is not that this is not important and I am sure it is very important to an individual that goes through this but in the greater scheme of things and in a resource-constrained environment you are going to choose the things that are most common that affect more people. That is the public health perspective. So unfortunately that is the harsh reality.” (MoH staff, Malawi)
	An external agenda	“This is not something that is discussed all the time and it has become important because someone somewhere has decided it is important.” (MoH staff, Malawi)
An important agenda	Need to inform patients about their health	“I feel it is very necessary because most people after completing their medication they actually do not know what next. I feel it would be very important so that they have some guidance on what else after the medication, what else after you get cured” (TB patient advocate, Kenya) “Let me speak it from my own point of view. Like for me, I would have found it more helpful if there were already systems in place because I would not have to feel like I am disturbing my surgeon with all those questions. I would have been more comfortable.” (TB-survivor, Kenya) “We do have a lot of patients. TB is quite a burden in Malawi so yes, it means every year we are discharging people from treatment so a lot of post-TB care candidates are coming out of our system each year and those need support and some level of attention to maintain their health in good check. So we do need to have that agenda.” (Researcher, Malawi) “So because on the cut-off date when they do the last tests and declare you free of TB, that is the end of the visitation, the end of going to the clinic, the end of everything. So they have left you with all the problems that you have been talking to them without getting any responses. So for me, I feel that it would have been important to still continue for some time so that they continue to monitor you and they come to a conclusion where you are also satisfied to say I have finished my treatment.” (TB Survivor, Malawi)
	Need for standardised patient pathways	**“**I think it is something that we should be advancing because patients are there and we need to have a unified approach to their care because now I believe they are being seen by different doctors everywhere who may make different diagnoses and manage them differently.” (Healthcare provider, researcher and lecturer, Kenya) **“…**we discussed with my colleagues within the department and at one point or another we refer these patients to the physicians who follow them up. From there we lose track of the patient. We do not have that mechanism of knowing how they have picked up from the consultant level.” (Healthcare Provider, Kenya) “That is why what we have done, we are liaising with our colleagues to say look, instead of saying NCD clinic, ART clinic, TB clinic, why don’t we just say a chronic disease clinic so that when a patient comes in, it is either we are giving them the TB drugs, they are getting ARTs, they are also getting the NCD drugs. In so doing, we will be able to manage these patients under one roof. So that is the integration that we want to have.” (MoH staff, Malawi) “As I said, if you look at TB, HIV and NCDs, I think this just needs to be one family because HIV patients they have TB, they develop NCDs so it is something that yes the three of us we have to be related. So yes, I would say it is good. It is something that needs to be done.” (MoH staff, Malawi)
	Opportunity to maximise the investment made during TB treatment	“Oh yes. It does not make sense that you invest millions in a person, you cure them and then after that they do not lead normal lives. I mean then what you have invested becomes a waste” (TB Patient advocate, Kenya) “I have observed that within one or two years the close contact of the same patients are coming to the hospital with the TB. So if we have a very well structured way of following up the post-TB cases, we can be able to pick the contacts early enough even without much of interruption or without them spreading to other people. So a timely agenda that if possible it needs to be in place”. (Non-governmental Organisation, Kenya)
	Means of reducing ongoing health seeking	“…I think [post-TB morbidity] is really rendering these people unproductive because they will spend a lot of time maybe seeking care, they will get all sorts of diagnosis and maybe put on all sorts of antibiotics or other medication and it is going to be expensive for them. It is going to drain their resources in time. From the healthcare system, I think it is also similar. It is a waste of resources because we are not giving these patients the care that they need maybe right from the time that they completed their TB treatment” (Multilateral Organisation–TB policy)

Post-TB morbidity was acknowledged by all participants as a real phenomenon experienced by TB-survivors. However, opinions about whether post-TB care should be prioritised within existing health care services were mixed (Table 2).

Management of post-TB morbidity was often framed as a form of individual care, and placed in opposition to public health interventions which operate at the population level and prevent transmission, and which must be prioritised within TB programmes. This perspective was seen amongst policy-makers and healthcare providers, and was related to beliefs about the role of national TB services. One researcher and two policy-makers felt that the focus on post-TB wellbeing was an external agenda, imposed by global researchers and policy-makers on countries, rather than being generated in country by National programmes.

*“There are more pressing issues*. *It is not that this is not important and I am sure it is very important to an individual that goes through this but in the greater scheme of things and in a resource-constrained environment you are going to choose the things that are most common that affect more people*. *That is the public health perspective*. *So unfortunately that is the harsh reality*.*”*

*(MoH staff*, *Malawi)*

In contrast, there were others who considered this a timely agenda, important to the health and wellbeing of TB-survivors. This was particularly the case amongst patient advocates who felt that post-TB morbidity should be addressed in order to allow patients to understand their own health, maximise their long-term wellbeing, and improve access to and ease of care.

*“I feel it is very necessary because most people after completing their medication actually do not know what next*. *I feel it would be very important so that they have some guidance on what else after the medication*, *what else after you get cured*.*”*

*(TB Patient advocate*, *Kenya)*

Healthcare providers spoke of the need to develop standardised pathways for post-TB care, highlighting challenges around unclear patient pathways, heterogeneity of current practice, and the pressure on individual providers to make clinical decisions in the absence of formal guidelines.

***“****I think it is something that we should be advancing because patients are there and we need to have a unified approach to their care*, *because now I believe they are being seen by different doctors everywhere who may make different diagnoses and manage them differently*.*”*

*(Healthcare provider*, *Kenya)*

Several participants felt that investing in recovery after TB disease would build on the investment already made in TB treatment, minimise ongoing health seeking by TB-survivors, reduce misdiagnoses of TB sequelae, and provide opportunities for contact screening and further counselling.

*“It does not make sense that you invest millions in a person*, *you cure them and then after that they do not lead normal lives*. *I mean then what you have invested becomes a waste”*

*(TB Patient advocate*, *Kenya)*

*“We do have a lot of patients*. *TB is quite a burden in Malawi so yes*, *it means every year we are discharging people from treatment so a lot of post-TB care candidates are coming out of our system each year and those need support and some level of attention to maintain their health in good check*. *So we do need to have that agenda*.*”*

*(Researcher*, *Malawi)*

### Structure and delivery of post-TB services (Table 3)

**Table 3:** Direct participant quotes: Structure and delivery of post-TB care services.

Theme	Sub-theme	Quotes
Content of post-TB services	Need for holistic care	“TB has very catastrophic effects and these patients end up being affected not just physically but also mentally. It has also a way of disabling their whole quality of life in totality…so I believe it is something we cannot ignore and it is something that we need to ensure that if we are talking about improving the quality of life of this particular TB patient, then we need to look at them holistically and ensure that they are being followed up beyond their period of treatment” (MoH staff, Kenya) “The consequences of post-TB complications is quite strenuous to the patient…because financially, emotionally, the patient is affected and even the normal health being of the patient is really affected. Nutrition, all those are affected. So if the donors are to be involved or the policy-makers, they should include all these people, nutritional, psychological counsellors such people that can help the patient holistically”. (Healthcare Provider, Kenya)
	Importance of broader management of TB-comorbidities	“And again we know that not only does TB cause obviously lung damage and longer term problems, but we also know that there are many other health conditions that contribute to a greater risk of someone developing TB, and those are often not treated and continue to pose ongoing risks for people. Whether it be things like smoking…or COPD….you know drinking alcohol” (Multilateral Organisation–TB policy)
	Importance of psychosocial support, and re-integration	“We forget that psychosocial support is supposed to be like a long term. We focus on it when somebody is on TB medication but medication is not only tablets…it is ensuring that we have proper counsellors or proper professionals that can provide psychosocial support even after treatment to ensure that somebody is well transitioned to the community”. (TB Patient advocate, Kenya) “…then the counselling for me is very very important and even training community counsellors as we have done for HIV. It would go a long way in supporting the TB-survivors.” (TB Survivor, Malawi) “It is a community issue, it is a social issue whether someone has lost their jobs, it is a social issue. Then you see it is also affecting many other aspects of development in terms of housing, in terms of education, in terms of you know livelihoods….[there is a need] to support those people who have had TB in terms of reintegration into the communities because it is not always physical. The impact is not always physical.” (TB Patient advocate, Malawi)
	Need for economic support	“… when you are transitioning from treatment, not everyone might need money but just to ensure that you have something like IGA–income generating activity” (TB patient advocate, Kenya) “…in terms of work, because some people cannot really do the work they used to do after TB. Maybe making sure people are getting vocational skills or maybe starting smaller businesses.” (TB Community Advocate, Malawi) “So we need to empower them. Economically, we have heard of cash transfer. I think those should be for the beneficiaries so that they can restart their life afresh.” (Healthcare provider, Malawi)
Location of post-TB services	Need for decentralisation, with a hierarchy of healthcare services	“The care should also be then provided in an affordable and accessible way closest to the patient. So perhaps the person with a mild presentation, we should be able to attend to them at the primary and even secondary level of care. So that only the persons who have very very severe (inaudible) with this condition would end up in the national referral or tertiary care or hospitals. That is how I would imagine it.” (Multilateral Organisation–TB Policy) “The trend in Malawi, the flow of patients, they will start from the primary healthcare which is the health centre, then secondary health facility like the district hospitals. If they fail there, they will go to the tertiary which is for the TB… Let them follow the same route because for most of the people, going to the tertiary it is expensive.” (Healthcare provider, Malawi)
	Need for community based care	“They need support and that is where we need civil society, community based organisations or patient support groups who can be linked to those people who are exiting the system that they are able to be integrated and supported to be reintegrated into the community.” (TB Patient advocate, Malawi)
	Importance of learning from HIV-NCD services	“….so initially you would start with high level cadres and then you need to learn as much as possible and develop detailed training, manuals and guidelines that are easy to follow. The HIV programme should be a good resource for whoever is working on this to look at how these rather complicated tasks were successfully handed over to lower cadres and how the services got as close to the patient as possible…and I would say start conservatively but do not take too long to decentralise.” (In country Researcher, Malawi) “In HIV first of all they all used to come in the tertiary hospitals because nobody knew how to handle it [HIV/AIDS]. But over time we developed our HIV systems, we developed systems protocols even. Now a patient does not need to walk very far. They can go to their nearest health centre and the nurse there has got very clear algorithms and she can manage to some level… So I think we have experience, we can learn lessons from the HIV programme and do it for lung health” (Healthcare Provider, Kenya)
Timing of post-TB services	Proactive care, from TB treatment completion	“It should be integrated from the beginning. Let us not wait for them to finish and then come back. In fact, I think if we would start treating this patient with that in mind that there is likely to be complications, there is even a way that we can be able to mitigate those complications. …” (TB Patient advocate, Kenya) “I think we need to be proactive but we should be proactive when we know what we will offer to these individuals. So screening or looking for individuals with the condition is not ethical if you are not able to provide care for them.” (Multilateral Organisation—TB Policy)
	Reactive care, responding to patient need	“The onus is on the patient that you feel a certain type of symptoms or whatever, and you will, the expectation is that you will have a health seeking behaviour to go to the hospital and say look I had TB and now I have noticed.” (Policy-maker, Malawi)
Delivery of post-TB services	Integration of services, for delivery	“We need to integrate with other services. The HIV services, the TB services, the Covid-19 services that are there…I would love if those activities can be integrated with other services that are already existing in that treatment level and prevention level and at community level. That could not make the work difficult to implement.” (MoH staff, Malawi) “TB programmes in most countries are not a vertical programme. Maybe [at the] national level, [or for] provincial level teams supervising the programmes and so on. But the service delivery level is integrated. I think that is what we need to promote…. I do not think we need to go for post-TB as another vertical approach. It should be integrated. It would make sense because we are talking about TB affecting other different organs and it is beyond TB now—it is post-TB. And that is where it makes sense to think about integration because the same person will have other problems. It could be HIV as well, and now we have COVID and post-COVID.” (Multilateral Organisation–TB Policy) “But I think there has to be a lot more emphasis about …the rest of the system to play its role. I think you will find that Ministries of Health loathe to verticalise things so much. So the thinking as I understand it is to allow the general health system to be able to deal with a wide swathe of problems rather than have specialised units or entities.” (Multilateral Organisation–TB policy)
Ownership / leadership of post-TB services	Leadership by the NTP, with support from others	“Who is responsible for it in Malawi, it would obviously be the Malawian health authorities, the NTP programme as far as I can see.” (Multilateral Organisation–TB policy) “I think more than ever, TB is slowly emerging to be a cross-cutting issue…so it is not something or an area that you would want to leave to TB program to solely address but they would obviously take lead…we need other players to come and support this…”(Non-governmental Organisation, Kenya)
	Challenges of NTP ownership	“I personally do not think TB programmes should be responsible for what happens after. We are a stakeholder but we are not the ones best equipped to deal with you if you are having difficulties breathing. That is not us. The people best equipped to do that would be clinical services and everything under clinical services.” (Policy-maker, Malawi) “Remember it is a National TB control programme so their priority is to control TB but not necessarily to rehabilitate. So it has to be carefully crafted to make sure that it does not look like an add-on to the TB programme but should be part of the TB programme.” (Multilateral Organisation–Funder) “The highlight on post-TB health I think is not new per se, but the emphasis is something that is new to a TB programme. So from a TB programme perspective it is simply designed to deal with the episode of the disease not what happens after” (Policy-maker, Malawi) “….they (NTLP) remain very lean and it will only be restricted to TB and leprosy as it is. So how do they even start to discuss that agenda of post-TB? Are they the right people to discuss that agenda or should they now move this discussion to the non-communicable disease side? So for me, to avoid a lot of back and forth, the easiest way is a lung health programme” (Healthcare Provider, Kenya) “It would be great if [ownership] was the TB programme. I cannot see that it would be responsible…there are so many different post-TB health issues that you could have… how does that work from an implementation standpoint?” (Multilateral Organisation–TB Policy)
	Challenges of situating within the health system	“For me it is outside the medical approach. So if we bring in another aspect that is outside the medical approach into the medical system then it will not be sustainable, it will not run. ….we need a structure that can be sustainable, that can make sure that they are carrying oversight and making sure that things are being done in the right order.” (TB Patient advocate, Malawi)
Framing of post-TB care, in discussion with patients	Need for careful communication	“…we are saying that TB is curable…So now if you say that after your medication there are complications that you can face, people now will be put off, to say what is going on? So I think it is a matter of in a way having clever programmatic and strategic programming because otherwise you might defeat the purpose …People still get cured of TB, but also we want to make sure that we are being realistic enough to say yes the medication might have side effects after but there is help…I think in a way it is a bit overwhelming because it feels like you are going to be a patient for the rest of your life.” (TB Patient advocate, Malawi)
	Presentation as part of the TB care cascade	“I think if it were to be like a continuation, people would take it more serious rather than when you are told now you have finished this treatment there is another phase because most people would be like why do I need it yet I feel I am okay. I think if it were to be one joint thing, it would work better” (TB Patient advocate, Kenya)
	Presentation as an optional service	“It should not be mandatory—‘Well you have finished TB we have got this package for you’…some others do not want to feel victimised and feel like they are victims of a certain thing.”(TB Patient advocate, Malawi)

#### Content of care

Although this study was initially framed around post-TB lung health, diverse stakeholders in both countries emphasized the need for multi-disciplinary post-TB services. This was a strong emerging theme. Dimensions of support identified included: health education, counselling and psychological support, nutritional support, social support, vocational training or financial support, and respiratory and physiotherapy services. Of note, discussions with international policy-makers were framed around the need to address broad TB comorbidities rather than just post-TB morbidity, including diabetes, HIV co-infection, smoking, alcohol use disorders, and chronic respiratory diseases such as chronic obstructive pulmonary disease (COPD).

Patient advocates expressed a strong feeling that post-TB morbidity should not be considered to be a purely medical problem, and that support should be provided in the community. Counselling and psychological support were perceived as a critical component, required both during and after TB treatment completion, given the long-term nature of TB related stigma, loss of livelihood, and residual post-TB physical morbidity. It was suggested that psychological support could be provided through individual or group counselling and support groups where people meet and share their experiences. The concept of TB patients being supported to ‘transition’ back into the community or ‘reintegrate’ after treatment completion was widely raised.

*“We forget that psychosocial support is supposed to be like a long term [issue]*. *We focus on it when somebody is on TB medication but medication is not only tablets…it is ensuring that we have proper counsellors or proper professionals that can provide psychosocial support even after treatment to ensure that somebody is well transitioned to the community”*.

*(TB Patient advocate*, *Kenya)*

It was noted that some TB-survivors may no longer be able to perform activities that they previously performed with ease, and that this might call for a change of profession. There was therefore a suggestion to provide such people with vocational skills training, and the potential for social protection in the form of cash transfers was also raised. Several participants suggested that government departments involved in the provision of social protection should be involved in discussions around holistic TB care.

#### Location of services

TB-survivors and advocates noted that post-TB services should be decentralised and provided to participants at the nearest health facility to ensure ease of access, and to allow for continuity of care after TB treatment completion. Several participants suggested that a hierarchy of support may be needed, with basic care provided at the primary care level, but more complex cases referred for specialist care. Several participants suggested that services should initially be developed and refined at the tertiary care level, and then decentralised. Opportunities to learn from the approach taken by HIV programmes for service development were often cited:

*“In HIV*, *first of all they all used to come in the tertiary hospitals because nobody knew how to handle it (HIV/AIDS)*. *But over time we developed our HIV systems*, *we developed systems protocols even*. *Now a patient does not need to walk very far*. *They can go to their nearest health centre and the nurse there has got very clear algorithms and she can manage to some level… So I think we have experience*, *we can learn lessons from the HIV programme and do it for lung health*.*”*

*(Healthcare Provider*, *Kenya)*

#### Timing of services

The majority of participants felt that post-TB services should be proactive in providing care–that is, aimed at identifying patients with morbidity at TB treatment completion, with ongoing support provided as part of the TB care cascade. This is the approach which has been outlined in the Kenyan NTLP guidelines. Early intervention was felt by community advocates to be important for preventing complications, optimising delivery of social support, and improving the uptake of services.

*“It should be integrated from the beginning*. *Let us not wait for them to finish and then come back*. *In fact*, *I think if we would start treating this patient with that in mind that there is likely to be complications*, *there is even a way that we can be able to mitigate those complications*. *…”*

*(TB Patient advocate*, *Kenya)*

However, this was not a universal perspective. Concerns were raised about the merits of adopting this proactive approach in the absence of evidence-informed clinical interventions which have been shown to improve patient outcomes. Some felt that the system should remain reactive, with responsibility for health-seeking if and when complications develop, remaining with TB-survivors:

*“The onus is on the patient that you feel a certain type of symptom or whatever*, *and you will*, *the expectation is that you will have a health seeking behaviour to go to the hospital and say*, *“look I had TB and now I have noticed”*.*”*

*(MoH staff*, *Malawi)*

#### Delivery of services

There was consensus that although post-TB services may be vertically led at the national and provincial level, the broad range of support required by TB-survivors will require an integrated approach at the service delivery level, with inclusion of TB and broader health and community services. Given the substantial proportion of TB patients with HIV co-infection in Kenya and Malawi, the need to integrate with HIV services both during and after TB treatment completion was highlighted, and opportunities to access long-term integrated care through HIV services were raised.

Conversations around who should deliver services were closely linked to discussions around responsibility and ownership of post-TB care services.

“*TB programmes in most countries are not a vertical programme*. *Maybe [at the] national level*, *[or for] provincial level teams supervising the programmes and so on*. *But the service delivery level is integrated*. *I think that is what we need to promote…*. *I do not think we need to go for post-TB as another vertical approach*. *It should be integrated*.*”*

(*Multilateral Organisation–TB policy*)

*“Instead of saying NCD clinic*, *ART clinic*, *TB clinic*, *why don’t we just say a chronic disease clinic so that when a patient comes in*, *it is either we are giving them the TB drugs*, *they are getting ARTs*, *they are also getting the NCD drugs*? *In so doing*, *we will be able to manage these patients under one roof*. *So that is the integration that we want to have*.*”*

*(MoH staff*, *Malawi)*

#### Ownership and leadership of services

There was some disagreement about the ownership and management of post-TB services at the national level. In Kenya, the post-TB agenda already sits within the NTLP. In Malawi, this had not been decided.

The majority of national and international participants felt that post-TB care should fall under the remit of NTPs to ensure continuity and consistency in service provision throughout the TB care cascade, and to build on existing resources and monitoring mechanisms. However, the need for broader integration with other programmes at the national level such as the division of NCDs, Ministry of Labour, Ministry of Gender, Social Security services, and implementing partners for service delivery was recognised. The need to position post-TB care as a core element of overall TB service delivery with this approach was highlighted.

*“I think more than ever*, *TB is slowly emerging to be a cross-cutting issue…so it is not something or an area that you would want to leave to TB program to solely address but they would obviously take lead…we need other players to come and support this…”*

*(Non-governmental organisation*, *Kenya)*

*“Remember it is a National TB control programme so their priority is to control TB but not necessarily to rehabilitate*. *So it has to be carefully crafted to make sure that it does not look like an add-on to the TB programme*, *but should be part of the TB programme*.*”*

(*Multilateral Organisation–TB policy*)

However, some caution about this approach was expressed by TB Programmes, healthcare providers, and patient advocacy groups. From the NTP perspective, a focus on post-TB wellbeing was felt to be outside of their remit, which historically has focused on the diagnosis and treatment of TB disease, with input ending at TB treatment completion. There was also a sense that the TB programme is not equipped to deal with the implementation of care for broader comorbidities on the ground, and that responsibility for post-TB service provision should therefore lie elsewhere. TB-survivors and patient advocates expressed some concern that positioning this agenda under the NTP would lead to a medical rather than a holistic approach to care.

*“I personally do not think TB programmes should be responsible for what happens after*. *We are a stakeholder but we are not the ones best equipped to deal with you if you are having difficulties breathing*. *That is not us*. *The people best equipped to do that would be clinical services and everything under clinical services*.*”*

*(MoH staff*, *Malawi)*

#### Framing of post-TB care

Patient advocates were keen that messaging on post-TB complications should be delivered carefully, emphasizing that TB is a curable disease, with a minority of TB patients developing residual morbidity, with services available for ongoing support. The need to ensure that TB-survivors are not unnecessarily defined as ‘patients’ or ‘victims’ of TB in the long-term was stressed. It was also felt that post-TB care should be presented to TB-survivors as optional, rather than a compulsory part of the TB care cascade:

*“…we are saying that TB is curable…So now if you say that after your medication there are complications that you can face*, *people now will be put off*, *to say what is going on*? *So I think it is a matter of having clever programmatic and strategic programming because otherwise you might defeat the purpose …People still get cured of TB*, *but also we want to make sure that we are being realistic enough to say yes the medication might have side effects after*, *but there is help…I think in a way it is a bit overwhelming because it feels like you are going to be a patient for the rest of your life*.*”*

*(TB Patient advocate*, *Malawi)*

*Barriers to implementation* (Table 4). Key barriers identified included the lack of local data on the burden of morbidity, limited funding, limited staff time, lack of equipment, and absence of clinical guidelines and models of care.

**Table 4:** Direct participant quotes: Barriers to implementation of post-TB care.

Theme	Sub-theme	Quotes
Lack of data	Local data on burden of disease	“That is the first thing we need to know—what is the magnitude of this, before we make a big deal of this. And I do not want to sound careless. I want you to understand this from priorities upon priorities for the system. Someone always gets left behind because you cannot fund everything. So you need to know first, how big a problem is this?” (Policy-maker, Malawi)
	Data on risk factors for morbidity	“But again, we need to understand the disease better and how it manifests itself. Is it the same here as in India, as in China or is it different depending whether you are a man or a woman, HIV positive or negative, on ART or not on ART, viral load suppressed or viral load not suppressed. Does it depend on your age? What does it depend on? Does it depend on the type of TB you had? If you had Xpert positive TB compared to perhaps clinically diagnosed TB or pleural effusion. I do not know if we have the answers to those questions so we cannot design the interventions until we know what we need to improve.” (Multilateral Organisation–TB Policy)
Funding constraints	Need for funding sources from outside the TB programme	“If you are saying well now countries should include longer term post-TB care in their national TB response … it will just probably receive a lot of pushback to say we already stretched enough…The win would need to be having this idea, this concept, bringing in other areas of funding to support it. I think that would make it much more palatable” (Multilateral Organisation–TB Policy)
	Need for donor funding	’Even drugs, drugs are being procured by the Global Fund, PEPFAR and World Bank and GDF of course which means if there is any support which is post-TB care… in my perspective it will definitely be donor driven and donor funded.” (TB Patient advocate, Malawi)
	Domestic funding options	“They need probably long term or lifelong support and you cannot expect that from a project or a grant with a shorter period of time. That is why a sustainable financing system should be in place and that is why this group should be prioritised as part of the overall health system and part of the domestic funding.” (Multilateral Organisation–Funder) “One is that if we make it (post-TB care) nationwide and we have an NHIF cover, then we can say that with NHIF you can go to the nearest facility that is near you. So people have the freedom… you can access that service in Nairobi so long as you have a code which is computerised. So with that code you are known that you are a regular recipient of this service so you can go to any facility near you to get that service.” (TB Patient advocate, Kenya)
	Need for mandate for care at the National/Global level, in order to secure funding	“So there should be a guidance from you know from WHO and international organizations and countries should also include this [post-TB] group in their national strategic pan and then they can still include some of the support to come from Global Fund.” (Multilateral Organisation—Funder) ’I think if it is in line with the WHO guidance I think it is something that we can always look positively but above all I think it has to be a national priority. If the NTP does not look at it as a priority I think it is unlikely that we are going to fund it. For example, the Global Fund this year allowed countries to develop their funding requests based on the National Strategic Plan (NSP). So if it is not in the NSP it is hard to fund it.” (Multilateral Organisation–TB policy)
Staffing constraints	Lack of trained staff	“The TB programme at the district level or facility level is not run by medically trained people. It is run by environmental health staff …while post-TB care would require doctors, nurses, clinical officers to do the work and there is not much interest especially from clinical officers and doctors to do TB work at facility level…” (Multilateral Organisation–Funder) “In so many of the countries that we work in there is huge lack of clinicians of trained [staff], whether it be very specialised services like radiologists or other clinical staff, even coming down to nurses. There is a huge gap in human resource capacity especially as we move out of the cities.” (Multilateral Organisation–Funder)
	Need for staff training	“What needs to be done is to train staff and let them know that these patients can have lung complications. I used to see for example some years back and this happens a lot at lower level health facilities when patients with TB who have completed treatment come back with symptoms. Often they have chest pains, they have fever, and they are put back on treatment again. Then they are treated fully again for another 6 months, 8 months, some even up to one year because their symptoms are not resolving. And this is simply because people have not done tests to find out what else could it be if this is not TB…” (Healthcare provider, Kenya)
Clinical guidelines	Need to define key clinical interventions which should be used	“Are there other health services, are there medicines, are there procedures that can be offered to them that would make their life better and respiratory function better? So would it be physiotherapy, some sort of pulmonary rehabilitation, education about these airway clearance exercises, regular immunizations against respiratory pathogens, definitely it will include smoking…” (Multilateral Organisation–Funder)
	Need to define key patient outcomes for monitoring & evaluation	“But having something around longer term health outcomes for people, there would have to be something that is achievable, measurable, something that could be impacted to I think to get funding and donors move around that cause.” (Multilateral Organisation–Funder)
Models of care	Lack of existing models of care	“Probably the second reason is that we have not seen really much happening in other countries either so if somebody can bring up a model that has worked elsewhere, I guess we should have an open mind to have a look at it and see how we can adapt that to our setup.” (Multilateral Organisation—Funder)
Potential impact of COVID-19	Greater need for integrated care	“It makes sense to think about integration because the same person will have other problems. It could be HIV as well. Now we have COVID and post-COVID and all those.” (Multilateral Organisation–Funder)
	Limited capacity for change	“I think our world is now going through these pandemic times and how exactly we will come out of this also financially and economically will determine at least in short and medium term how the world and how all of us can take up new issues or newer issues. Because you know every organisation, every human being has some sort of saturation point I think.”(Multilateral Organisation–TB Policy)

#### Limited data

Athough many study participants felt that post-TB morbidity was a common problem, there was a broad sense that more in-country data are needed to describe the burden of this morbidity, and risk factors for disease, in order to inform decision making around investment by NTPs and international funders.

*“That is the first thing we need to know—what is the magnitude of this*, *before we make a big deal of this*. *And I do not want to sound careless*. *I want you to understand this from priorities upon priorities for the system*. *Someone always gets left behind because you cannot fund everything*. *So*, *you need to know first*, *how big a problem is this*?*”*

*(MoH staff*, *Malawi)*

#### Funding constraints

The lack of secure funding for post-TB care was highlighted as a key barrier to care. Given the heavy reliance of Kenya and Malawi on donor funding for TB service delivery, many participants felt that donor funding would be needed to support post-TB services. There was some discussion of whether this should be requested within the TB envelope, or accessed through non-TB funding streams. The main funder for TB services in Kenya and Malawi is The Global Fund, but neither country has included post-TB care in their funding requests submitted to The Global Fund to date. There were a range of perspectives on whether The Global Fund would be responsive to such requests from individual countries, or whether guidelines from external bodies such as the World Health Organization would first be required.

Other participants suggested that post-TB care should be funded by the government to ensure sustainability, with a broad range of options for domestic funding raised, including health insurance programmes such as the National Health Insurance Framework (NHIF) in Kenya, disaster relief schemes, social protection programmes, and pension schemes.

#### Staff and equipment constraints

Concern was raised about the human resources needed to deliver post-TB support, particularly using a decentralised approach and in rural areas. Although use of spirometry and chest X-ray are suggested at TB treatment completion within the Kenyan NTLP guidelines, it is noted that access to this equipment in the majority of healthcare facilities is limited. The need for capacity building for post-TB care was expressed.

*“The TB programme at the district level or facility level is not run by medically trained people*. *It is run by environmental health staff …while post-TB care would require doctors*, *nurses*, *and clinical officers to do the work*, *and there is not much interest especially from clinical officers and doctors to do TB work at the facility level…”*

(*Multilateral Organisation–Funder*)

#### Need for clinical guidelines and models of care

The lack of robust clinical guidelines for the diagnosis and management of post-TB complications, lack of existing models of care, and the need for clear patient outcomes which could be used for monitoring and evaluation of interventions were identified as key barriers to implementation.

*“Probably the second reason is that we have not seen really much happening in other countries either*. *So if somebody can bring up a model that has worked elsewhere*, *I guess we should have an open mind to have a look at it and see how we can adapt that to our setup*.*”*

(*Multilateral Organisation—Funder*)

*“But having something around longer term health outcomes for people*, *there would have to be something that is achievable*, *measurable*, *something that could be impacted to I think to get funding and donors move around that cause*.*”*

(*Multilateral Organisation—Funder*)

Although the impact of the COVID-19 pandemic was not explicitly explored in this study, this was raised as a factor which might either expedite the introduction of more integrated models of respiratory care, or act as a barrier to further change.

## Discussion

Our understanding of the burden and nature of post-TB morbidity is growing, and clinical guidelines for post-TB care are under development [18, 20]. However, little work has been done to explore how integrated post-TB services might be delivered by health systems on the ground, particularly in resource-constrained settings. In this study we explored stakeholder perspectives on post-TB care in Kenya and Malawi, including discussion with patient advocates, healthcare providers, policy-makers, national TB and NCD programmes, and funders. Our work has highlighted key challenges including the need for broad multidisciplinary services, uncertainty about governance and leadership, lack of local data on the burden of morbidity, lack of funding, and concerns about staff and equipment capacity. However, the study has also demonstrated widespread interest in this agenda, identified key research opportunities, and highlighted opportunities to learn from HIV-services.

There was broad consensus amongst stakeholders that TB-patients and TB-survivors require holistic care, which addresses the physical, social, psychological, and economic impacts of TB disease. Several international stakeholders emphasised the need to address TB-comorbidities such as smoking, diabetes, and alcohol use disorders alongside post-TB morbidity, to support long-term patient wellbeing, in keeping with existing international guidelines [21, 22]. The importance of addressing TB multimorbidity has been highlighted elsewhere [23–25]. While our initial focus in this study was the management of post-TB lung disease, it is clear that a larger conversation around the delivery of integrated patient-centred care across the TB cascade–including both comorbidities and post-TB sequalae–is needed.

However, there is considerable uncertainty over how this broad holistic care should be managed and delivered. Many stakeholders felt that post-TB care should fall under the remit of NTPs. This would allow continuity of care across the TB care cascade, and would leverage existing systems for the delivery and monitoring of services. Indeed, several pilot programmes of integrated patient care for TB comorbidities such as diabetes and smoking have employed this approach, building off NTP frameworks [26]. However, there was some hesitancy from NTPs to take on this responsibility, alongside their role in the diagnosis and treatment of active TB disease. This may relate to concern about funding, staff time, and expertise, but is also rooted in the belief that the management of comorbidities falls outside of the traditional remit of TB services. If we are to use an NTP-centred approach to post-TB care, the management of comorbidities and sequelae will need to be framed as an integral part of the TB care cascade, and stronger links will be needed between TB and NCD programmes, both at the national-level for governance and leadership, and within health facilities for service delivery [27]. Links with community services and peer support networks may be required, particularly for the delivery of psychosocial support [28].

Operational research will also be needed to understand how TB patients might move between TB, NCD and community-based services. The development of models of care could be informed by existing approaches to the delivery of integrated HIV services in resource-constrained settings [29–31]. Given that approximately 25% of TB patients in Kenya and 45% in Malawi are HIV co-infected [32], with many TB-survivors continuing to engage with HIV services even after TB treatment completion, direct integration with HIV services could be explored. Whatever the approach used, careful evaluation will be needed to determine the cost, impact, barriers and facilitators of implementation, and generalisability of these models of care, and existing guidelines for service development and evaluation should be used in pilot programmes where possible [33–35].

Our study has highlighted several data gaps which must be addressed to inform decision making by funders and policy-makers around investment in post-TB care. These include the need for in-country, local level data on the burden and distribution of post-TB morbidity. Low cost approaches to generating these data should be explored, including routine surveillance of post-TB morbidity amongst TB survivors by National TB programmes, and the use of modelling work. Funders have highlighted the need for clear metrics through which the impact of post-TB services could be evaluated, and international groups currently developing clinical guidelines for the management of post-TB morbidity are well positioned to develop these metrics.

However, our data also suggest some circularity in decision making for the support of post-TB care: NTPs may feel unable to advocate for post-TB care due to the lack of funding and lack of WHO guidelines, but funders and the WHO may require member countries to declare this as a priority area in order to move forward. Further work with policy-makers and funders may help to break this impasse. Advocacy for TB-patients and survivors will be needed to support this work, and our data and that of others suggest that the patient voice may be particularly powerful here [16].

### Strengths and limitations

This study has several limitations. It was designed as a programme of stakeholder engagement rather than primary qualitative research. As such, we did not reach full saturation in our data, and there may have been some bias in both the questions asked and the responses received during data collection. The snowballing approach will likely have identified individuals who are active or influential in this space, but may have missed less powerful individuals. COVID-19 related constraints on time and travel meant that patients and providers from rural areas, and front-line health workers were under-represented. We were largely constrained to one-to-one engagements, with limited opportunity to explore differences in perspectives in group discussions in detail. Lastly, we note the perspective raised by some stakeholders that the post-TB agenda is an external agenda, being imposed from outside. Whilst this was a minority perspective, we are grateful that participants felt comfortable to raise this concern, and acknowledge that this may have shaped some of the discussions held during data collection. This is a reminder that any efforts to take the post TB care agenda forward should be driven by local priorities, buy-in and leadership.

This work also has several strengths. This study is novel in its focus on how post-TB services might be delivered on the ground in LMIC settings. Despite calls for post-TB care, questions about practical implementation within existing health and community services remain largely unanswered. Our findings about the leadership, content and models of care will be critical to the development of pilot studies of post-TB services in Kenya and Malawi, and possibly other African countries with similar disease profiles and health systems. More broadly, our approach highlights the value of formative stakeholder engagement work in defining local priorities and challenges, prior to health system interventions. The study has strengthened the network of local stakeholders with influence and interest in this area, who are well positioned to take this agenda forward.

## Conclusion

Although international TB guidelines and policies remain focused on the management of TB disease, our findings highlight a need for holistic care, which addresses the broad physical, social, psychological, and economic wellbeing of TB patients, across the TB care cascade, including the post-TB period. Delivering patient-centred care in this way will require stronger collaboration between TB and non-communicable disease programmes at the national level, and health facility and community services at the local level. This study highlights key evidence gaps which must be addressed to support decision making by funders and policy-makers, identifies challenges which must be considered prior to implementation, and suggests opportunities to learn from integrated HIV services. Although this work was focused on Kenya and Malawi, inclusion of international partners including the Stop TB Partnership, The Global Fund and The Union has provided some global perspectives that go beyond the Eastern Africa region. We believe these data and the strong networks built through this work provide strong foundations for the development and evaluation of pilot studies of integrated TB care.

## Supporting information

S1 File (DOCX)

S1 Checklist Standards for Reporting Qualitative Research (SRQR) checklist. (DOCX)

## References

[1] World Health Organisation. Global tuberculosis report 2021. Geneva, Switzerland: World Health Organisation, 2021.

[2] Dodd PJ, Yuen CM, Jayasooriya SM, van der Zalm MM, Seddon JA. Quantifying the global number of tuberculosis survivors: a modelling study. *The Lancet Infectious Diseases* 2021; **Online ahead of print**. doi: 10.1016/S1473-3099(20)30919-1 33640076

[3] Meghji J, Lesosky M, Joekes E, Banda P, Rylance J, Gordon S et al. Patient outcomes associated with post- tuberculosis lung damage in Malawi: a prospective cohort study. *Thorax* 2020; 0: 1–10. doi: 10.1136/thoraxjnl-2019-213808 32102951 PMC7063395

[4] Meghji J, Simpson H, Squire SB, Mortimer K. A Systematic Review of the Prevalence and Pattern of Imaging Defined Post-TB Lung Disease. *PloS one* 2016; 11(8): e0161176. doi: 10.1371/journal.pone.0161176 27518438 PMC4982669

[5] Amaral AF, Coton S, Kato B, Tan WC, Studnicka M, Janson C, et al. Tuberculosis associates with both airflow obstruction and low lung function: BOLD results. *Eur Respir J* 2015; 46(4): 1104–12. doi: 10.1183/13993003.02325-2014 26113680 PMC4594762

[6] Ravimohan S, Kornfeld H, Weissman D, Bisson GP. Tuberculosis and lung damage: from epidemiology to pathophysiology. *Eur Respir Rev* 2018; 27(147). doi: 10.1183/16000617.0077-2017 29491034 PMC6019552

[7] Meghji J, Gregorius S, Madan J, Chitimbe F, Thomson R, Rylance J et al. The long term effect of pulmonary tuberculosis on income and employment in a low income, urban setting. *Thorax* 2020; 76(4): 387–95. doi: 10.1136/thoraxjnl-2020-215338 33443228 PMC7982936

[8] Allwood BW, van der Zalm MM, Amaral AFS, Byrne A, Datta S, Egere U, et al. Post-tuberculosis lung health: perspectives from the First International Symposium. *Int J Tuberc Lung Dis* 2020; 24(8): 820–8. doi: 10.5588/ijtld.20.0067 32912387

[9] Romanowski K, Baumann B, Basham CA, Ahmad Khan F, Fox GJ, Johnston JC. Long-term all-cause mortality in people treated for tuberculosis: a systematic review and meta-analysis. *The Lancet Infectious diseases* 2019; 19(10): 1129–37. doi: 10.1016/S1473-3099(19)30309-3 31324519

[10] Ranzani OT, Rodrigues LC, Bombarda S, Minto CM, Waldman EA, Carvalho CRR. Long-term survival and cause-specific mortality of patients newly diagnosed with tuberculosis in São Paulo state, Brazil, 2010–15: a population-based, longitudinal study. *The Lancet Infectious Diseases* 2020; 20(1): 123–32. doi: 10.1016/S1473-3099(19)30518-3 31676242 PMC6928568

[11] Marx FM, Floyd S, Ayles H, Godfrey-Faussett P, Beyers N, Cohen C. High burden of prevalent tuberculosis among previously treated people in Southern Africa suggests potential for targeted control interventions. *Eur Respir J* 2016; 48(4): 1224–7. 27390274 10.1183/13993003.00716-2016 PMC5512114

[12] Marx FM, Dunbar R, Enarson DA, Williams BG, Warren RM, van der Spuy GD et al. The temporal dynamics of relapse and reinfection tuberculosis after successful treatment: a retrospective cohort study. *Clin Infect Dis* 2014; 58(12): 1676–83. doi: 10.1093/cid/ciu186 24647020

[13] Menzies NA, Quaife M, Allwood BW, Byrne A, Coussens AK, Harries AD, et al. The lifetime burden of disease due to incident tuberculosis: a global re-appraisal including post-TB sequelae. *The Lancet Global health* 2021. 10.1016/S2214-109X(21)00367-3 PMC8609280 34798027

[14] Tomeny EM, Nightingale R, Chinoko B, Nikolaidis GF, Madan J, Worrall E, et al. TB DALYs overlook the contribution of post-TB disability: evidence from urban Malawi. 2021. 10.1136/bmjgh-2021-007643 PMC9125716 35606014

[15] Harries AD, Dlodlo RA, Brigden G, Mortimer K, Jensen P, Fujiwara PI, et al. Should we consider a ’fourth 90’ for tuberculosis? *Int J Tuberc Lung Dis* 2019; 23(12): 1253–6. doi: 10.5588/ijtld.19.0471 31753065

[16] Schoeman I, Sifumba Z. Tuberculosis care does not end at treatment completion—a perspective from tuberculosis survivors. *The Lancet Infectious Diseases* 2021; **Online ahead of print**. 10.1016/S1473-3099(20)30941-5 33640078

[17] Allwood B, van der Zalm M, Makanda G, Mortimer K, Amaral AFS, Egere U, et al. The long shadow post-tuberculosis. *The Lancet Infectious Diseases* 2019; 19(11): 1170–1. doi: 10.1016/S1473-3099(19)30564-X 31657778

[18] Migliori GB, Marx FM, Ambrosino N, Zampongna E, Schaaf HS, van der Zalm MM, et al. Clinical standards for the assessment, management and rehabilitation of post-TB lung disease. *Int J Tuberc Lung Dis* 2021; 25(10): 797–813. doi: 10.5588/ijtld.21.0425 34615577 PMC8504493

[19] Mungai BN, Joekes E, Masini E, Obasi A, Manduku V, Mugi B, et al. ’If not TB, what could it be?’ Chest X-ray findings from the 2016 Kenya Tuberculosis Prevalence Survey. *Thorax* 2021. 10.1136/thoraxjnl-2020-216123 PMC8223623 33504563

[20] Allwood B, Meghji J. Management of Tuberculosis: A guide to essential practice. 7th Edition. Paris, France: The International Union against Tuberculosis and Lung Disease, 2019.

[21] World Health Organisation Disease TIuaTaL. Collaborative framework for care and control of tuberculosis and diabetes. Geneva, Switzerland: World Health Organisation, 2011. 26290931

[22] World Health Organisation. A WHO / The Union monograph on TB and tobacco control: joining efforts to control two related global epidemics. Geneva, Switzerland: World Health Organisation, 2007.

[23] Siddiki K, Stubbs B, Lin Y, Elsey H, Siddiki N. Tuberculosis Multimorbidity: A Global Health Challenge Demanding Urgent Attention. *Int J Tuberc Lung Dis* 2021; 25(2): 87–90. 33656417 10.5588/ijtld.20.0751

[24] Marais BJ, Lönnroth K, Lawn SD, Migliori GB, Mwaba P, Glaziou P, et al. Tuberculosis comorbidity with communicable and non-communicable diseases: integrating health services and control efforts. *The Lancet Infectious Diseases* 2013; 13(5): 436–48. doi: 10.1016/S1473-3099(13)70015-X 23531392

[25] Meghji J, Mortimer K, Mpangama S. TB and COPD in low-income settings: a collision of old foes. *Thorax* 2021. doi: 10.1136/thoraxjnl-2021-218220 34853155

[26] Li L, Lin Y, Mi F, Tan S, Liang B, Guo C, et al. Screening of patients with tuberculosis for diabetes mellitus in China. *Tropical Medicine and International Health*; 17(10): 1294–301. doi: 10.1111/j.1365-3156.2012.03068.x 22830945

[27] Workneh MH, Bjune GA, Yimer SA. Assessment of health system challenges and opportunities for possible integration of diabetes mellitus and tuberculosis services in South-Eastern Amhara Region, Ethiopia: a qualitative study. *BMC Health Serv Res* 2016; 16: 135. doi: 10.1186/s12913-016-1378-6 27095028 PMC4837556

[28] Khanal S, Elsey H, King R, Baral SC, Bhatta BR, Newell JN. Development of a Patient-Centred, Psychosocial Support Intervention for Multi-Drug-Resistant Tuberculosis (MDR-TB) Care in Nepal. *PloS one* 2017; 12(1): e0167559. doi: 10.1371/journal.pone.0167559 28099475 PMC5242498

[29] Kemp CG, Weiner BJ, Sherr KH, Kupfer LE, Cherutich PK, Wilson D, et al. Implementation science for integration of HIV and non-communicable disease services in sub-Saharan Africa: a systematic review. *AIDS (London*, *England)* 2018; 32 Suppl 1: S93–s105. doi: 10.1097/QAD.0000000000001897 29952795

[30] Njuguna B, Vorkoper S, Patel P, Reid MJA, Vedanthan R, Pfaff C, et al. Models of integration of HIV and noncommunicable disease care in sub-Saharan Africa: lessons learned and evidence gaps. *AIDS (London*, *England)* 2018; 32 Suppl 1(Suppl 1): S33–s42. doi: 10.1097/QAD.0000000000001887 29952788 PMC6779053

[31] Vorkoper S, Kupfer LE, Anand N, Patel P, Beecroft B, Tierney WM, et al. Building on the HIV chronic care platform to address noncommunicable diseases in sub-Saharan Africa: a research agenda. *AIDS (London*, *England)* 2018; 32 Suppl 1(Suppl 1): S107–s13. 29952796 10.1097/QAD.0000000000001898 PMC6438180

[32] World Health Organisation. Global Tuberculosis Programme: Tuberculosis country, regional and global profiles. 2021. https://www.who.int/teams/global-tuberculosis-programme/data (accessed 13th December 2021.

[33] O’Cathain A, Croot L, Duncan E, Rousseau N, Sworn K, Tuner KM, et al. Guidance on how to develop complex interventions to improve health and healthcare. *BMJ Open* 2019; 9(8): e029954. doi: 10.1136/bmjopen-2019-029954 31420394 PMC6701588

[34] Hoffmann TC, Glasziou PP, Boutron I, Milne R, Perera R, Moher D, et al. Better reporting of interventions: template for intervention description and replication (TIDieR) checklist and guide. *BMJ* 2014; 348: g1687. doi: 10.1136/bmj.g1687 24609605

[35] Kirk MA, Kelley C, Yankey N, Birken SA, Abadie B, Damschroder L. A systematic review of the use of the Consolidated Framework for Implementation Research. *Implement Sci* 2016; 11: 72. doi: 10.1186/s13012-016-0437-z 27189233 PMC4869309

