# Supplementary material for: Stakeholder perspectives around post-TB wellbeing and care in Kenya and Malawi
Source: PLOS Glob Public Health. 2022 Sep 7;2(9):e0000510. doi: 10.1371/journal.pgph.0000510 (PMC10022351; doi:10.1371/journal.pgph.0000510)
Supplement: S1 File — (DOCX) [file pgph.0000510.s001.docx]

**Appendices**

**Stakeholder perspectives around post-TB wellbeing and care in Kenya and Malawi**

Sarah Karanja, Tumaini Malenga, Jessie Mphande, Stephen Bertel Squire, Jeremiah Chakaya Muhwa, Ewan M Tomeny, Laura Rosu, Stephen Mulupi, Tom Wingfield, Eliya Zulu, Jamilah Meghji

**Appendix A: Data collection tools**

A summary data collection tool used is given here. Different combinations of questions were used for the different stakeholder groups (TB survivors / patient advocates, Local and regional stakeholders, international stakeholders) to reflect their different experience, but the broad categories covered remained the same. The questions included in interviews evolved over the duration of the study.

| **Category of questions** | **Questions / probes** |
| --- | --- |
| **General Introduction** | **We would like to understand what aspects of TB care you and your organisation work on.** |
|  | What is the role of your organisation, with regards to TB care?  What is your role within the organisation? |
| **Understanding of post-TB morbidity** | **We would like to understand your thoughts about what happens to people after they finish TB treatment.** |
|  | From your experience what happens to people after completing TB treatment? / What do you know about people’s lives and wellbeing after TB?  *Probe: What is believed to be known / not known*  *Probe: How is this known*  Where have you found out about people’s lives after TB?  *Probe: Experience, sources of information*  Do TB patients faced any ongoing issues or challenges when they have finished TB treatment?  *Probe: Physical health, Mental health, Social issues, Economic wellbeing*  What is your understanding about post-TB lung health specifically?  *Probe: What is believed to be known / not known*  *Probe: How is this known* |
| **Previous experience** | **We would like to ask about previous experience you or your organisation may have had, working in post-TB care** |
|  | Have you/your organisation been involved in providing care or services to patients after TB treatment in the past? What do you think about this?  *Probe – details of involvement, perspectives on involvement / lack of involvement*  Have there been any discussions around introducing post-TB care within your organisation?  *Probe: What was discussed, with whom, outcome*  Does this area fit with the overall priorities of your organisation? What are these priorities?  Why do you think this is / is not an area of focus for your organisation? |
| **Post-TB agenda** | **We would like to ask you a bit more about your thoughts on this area of post-TB wellbeing and care** |
|  | For you, at the moment, what would you say are the most important aspects of looking after TB Patients? What in your opinion are the main priorities?  What do you think about this area of work, looking at the wellbeing of patients who have been treated for TB disease?  Is this an area which is relevant to your work, or the work your organisation does?  What do you think, in general, about trying to support TB patients after they finish TB treatment?  *Probe: why / why not?*  *Probe: different types of support – physical, mental health, social, economic*  Do you think this is something we should be addressing?  *Probe: why / why not?*  *Probe: Concerns about the agenda*  Why do you think people are starting to discuss this area now – how do you feel about this? |
| **General facilitators / barriers** | **We would like to understand a little about other facilitators or barriers to post-TB care** |
|  | Why is post-TB care not something that has been or is being done?  What would be the main barriers to introducing a package of post-TB care?  How could we address these barriers?  Are there any facilitators which could support a post-TB program?  How could we make the most of these facilitators?  What might encourage introducing post-TB care going forward?  What capacity, if any, do you think we would need to build, in order to introduce post-TB services? |
| **Leadership & governance** | **We would like to understand your perspectives on who might be responsible for designing and delivering post-TB care in country** |
|  | Who do you think would or should be responsible for post-TB care services, if these were introduced?  What should the role of the National TB Programme be?  What should the role of other parts of the health system be?  What is the current relationship between the NTP and NCD departments?  *Probe: Links NCD and TB, NCD and HIV, examples of integrated care*  *Probe: What does / doesn’t work* |
| **Financial resources** | **We would like to understand what the funding situation might be like, with respect to post-TB care.** |
|  | If we were to introduce post-TB care in say Kenya/ Malawi, how do you think this might be funded?  Are there certain organisations or donors who you think could or should support this, through funding?  Who makes decisions around how TB care, and post-TB care, might be funded?  *Probe: regional level and global?* |
| **Contents of post-TB care** | **We would like to understand what you think post-TB care services might include** |
|  | If we were to develop a of ongoing care, to support people after TB treatment completion, what services should it provide? *Probe – different aspects of morbidity to address, interventions, why*  If we were to develop a package of post-TB care, what are the most important outcomes which we should be trying to improve?  *Probe – Clinical outcomes for patient, health system outcomes*  Are there certain interventions which you are particularly interested in, or which you think researchers should prioritise looking at? |
| **Key patient groups** | **We would like to understand your thoughts about who should receive ongoing support after TB treatment completion** |
|  | Who do you think should be offered support after completing TB treatment?  *Probe – everyone or just a select group, which groups, why?*  If we only offer support to some groups, who should these groups be?  *Probe – who and why?, how to identify?* |
| **Structure of post-TB care** | **We would like to understand what you think post-TB care services might look like** |
|  | What are your recommendations on service provision?  How should post-TB care be delivered?  If we were to introduce a package of post-TB care, who should be responsible for delivering it? *Probe – different health care providers, peers, community workers*  *Probe – pros / cons*  Where should services be delivered?  *Probe – health facilities or the community Probe – If in health facilities, decentralised or centrally*  *Probe – pros / cons*  Should support services for those completing TB treatment be embedded within routine hospital care, or kept separate?  *Probe – why, pros & cons*  Do you think we should be pro-active in delivering post-TB care, or reactive? That is – should we be trying to identify people with residual morbidity after treatment and following them up, or should we discharge patients and let them come back if they are unwell afterwards?  *Probe – why, pros & cons* |
| **Decision making around investment and implementation** | **We would like to understand how decisions about post-TB care might be made.** |
|  | How are decisions made, for issues like this to be prioritised?  *Probe: Who makes the decision, any key meetings and forums*  Do you feel that we have enough information about post-TB wellbeing to make decisions about post-TB care?  What additional information would help to inform these decisions?  *Probe: Specific data needs, why needed*  How should this knowledge be generated?  *Probe: By who – which institutions/ organisations should lead this*  *Probe: Where – in country, form the region, from other parts of the world*  If we as a TB community decided that this is a set of services we wish to provide – how would we work towards this?  Who would be needed to champion this at the international level? |
| **TB survivor needs**  **(Asked to TB advocates / survivors)** | **Our team has been thinking about how we might support TB survivors after TB treatment completion. We would like to understand your thoughts on this.** |
|  | If someone said to you that they were setting up a service to support patients completing TB treatment, what should the service do, differently from the existing TB care services?  *Probe– anything which would have helped you, after you finished your treatment?*  *Probe – information / education required*  *Probe – specific types of support needed with health / social / financial support*  What would be important to TB survivors, in deciding whether to use a service like this?  *Probe: facilitators / barriers to use*  *Probe: Confidentiality, cost, distance, timing & frequency of appointments, who delivers care*  Where should they receive this support, after TB treatment completion?  *Probe: Health centre, community, other*  Who should provide this service?  *Probe – TB services or not, why / why not?*  Is it important for TB survivors to continue to see someone regularly, after finishing TB treatment?  *Probe - why / why not?*  Where do patients go if they became unwell again, with chest symptoms or TB symptoms like last time?  *Probe - Would they go back to the TB Officers? Why / why not? Probe - Would they prefer to see someone else? Why / why not? Who?*  What would stop patients from going to see a health care provider, if they became unwell again?  *Probe – Why / why not?*  How could we make it easier for them to see a health care provider, if they became unwell again after TB treatment?  *Probe – barriers and facilitators of health seeking* |
| **Final section** | **Thanks and suggestions** |
| **Other stakeholders** | Are there other people who are interested in this area, who you think we should speak too, to understand their perspectives? Inside or outside your organisation?  Are there other people who are influential in decision making, who we should speak with? |
| **Other areas** | Are there any other areas which you think are relevant to post-TB care, which we have not discussed?  Do you have any questions for us? |

**Appendix B:** Coding framework

| Category | Item | Sub-code |
| --- | --- | --- |
| Knowledge about post-TB sequelae | Conceptualisation of TB disease |  |
|  | Generation of knowledge |  |
|  | Knowledge of post-TB morbidity |  |
|  | Knowledge of post-TB lung disease |  |
| Barriers to introducing post-TB care | Need for local data |  |
|  | Need for data in general |  |
|  | Responsibility for collecting data |  |
|  | Not a priority |  |
|  | Structure - medical approach |  |
|  | Historical remit of TB services |  |
|  | Beliefs re. size of problem / need |  |
|  | Lack of existing models of care |  |
|  | Need for evidence-based interventions |  |
|  | Need for guidelines and policies |  |
| Barriers to health system delivery | Funding constraints |  |
|  | Equipment limitation |  |
|  | Health service constraints |  |
|  | Capacity building |  |
|  | Health Management Information System |  |
|  | Sustainability |  |
|  | Weak or non-agile health systems |  |
|  | Infrastructure |  |
|  | Political will |  |
|  | Staffing constraints |  |
| Patient barriers to post-TB care | Do not want to keep attending facilities |  |
|  | Indirect cost |  |
|  | Lack of patient-centred services |  |
|  | Livelihood |  |
|  | Stigma |  |
|  | Difficult access to care |  |
| Structure of post-TB care | Ownership & responsibility |  |
|  | Need for decentralised care |  |
|  | Other |  |
|  | Proactive or reactive |  |
|  | Targeting patients |  |
|  | Need for specialist referral |  |
|  | Comments re. structure of care | Where Who should get care Who should provide care |
|  | Approaches to M&E |  |
| Content of post-TB care | Priorities for post-TB care |  |
|  | Health education and information |  |
|  | Mental health |  |
|  | Other |  |
|  | Physiotherapy |  |
|  | Management of multimorbidity |  |
|  | Social support |  |
|  | Respiratory services |  |
|  | Vocational support |  |
|  | Nutritional support |  |
| Means of delivering care | Task shifting |  |
|  | Health Surveillance Assistants or Community Health Volunteers |  |
|  | Integration |  |
|  | Patients as providers |  |
| Decision making for post-TB care | Prioritisation |  |
|  | Public health vs. clinical care |  |
|  | Patients as advocates |  |
|  | Advocacy |  |
|  | Existing priorities in TB care |  |
|  | Decision making pathways |  |
| Attitudes towards agenda | PTLD as an external agenda |  |
|  | Beliefs about agenda | Improve health Lack of structured follow up Maximize investment Neglected Opportunity to monitor those with complications Other Priority or not priority |
|  | Timing of agenda |  |
|  | Suggestions for research going forward | Planned or ongoing research Other |
|  | Not systematic |  |
|  | Broaden the scope beyond medical care |  |
|  | No discussion around post-TB care |  |
| Funding options | MOH |  |
|  | Other funders |  |
| TB survivors experience | Attitude of health care providers |  |
|  | Financial hardship |  |
|  | Mental wellbeing |  |
|  | Nutrition support |  |
|  | Physical wellbeing |  |
|  | Stigma |  |
